# Neighborhood Context, Homeownership and Home Value: An Ecological Analysis of Implications for Health

**DOI:** 10.3390/ijerph14101098

**Published:** 2017-09-22

**Authors:** Roshanak Mehdipanah, Amy J. Schulz, Barbara A. Israel, Graciela Mentz, Alexa Eisenberg, Carmen Stokes, Zachary Rowe

**Affiliations:** 1 School of Public Health, University of Michigan, Ann Arbor, MI 48109, USA; ajschulz@umich.edu (A.J.S.); ilanais@umich.edu (B.A.I.); gmentz@umich.edu (G.M.); alexae@umich.edu (A.E.); 2 School of Nursing, University of Detroit Mercy, Detroit, MI 48221, USA; stokesla@udmercy.edu; 3 Friends of Parkside, Detroit, MI 48213, USA; zrowe@friendsofparkside.org

**Keywords:** homeownership, health inequities, Detroit Metropolitan Area, housing value, race, disability, mortality

## Abstract

While homeownership has been linked to positive health outcomes there is limited evidence regarding the conditions under which it may be health protective. We present a conceptual model linking homeownership to health, highlighting key potential pathways. Using the Detroit Metropolitan Area as a case study, and data from the American Community Survey (2009–2013; 5-years estimates) and Michigan Department of Community Health, we tested the following questions: (1) Is neighborhood percentage non-Hispanic Black (NHB) associated with homeownership? (2) Is neighborhood percentage NHB associated with health? (3) Is the association between percentage NHB and health mediated by homeownership? (4) Does neighborhood housing value modify associations between percentage NHB and health, or between homeownership and health? Percentage NHB was associated with homeownership and health outcomes; Associations between percentage NHB and mortality, but not disability, were partially mediated by neighborhood homeownership. Neighborhood housing value modified associations between neighborhood homeownership and both disability and mortality, but not between percentage NHB and health outcomes. Findings are consistent with the thesis that health-promoting effects of homeownership may be contingent upon house values. These results add to a limited body of evidence suggesting that variations in homeownership may contribute to persistent racial and socioeconomic health inequities.

## 1. Introduction

Existing literature connects homeownership to positive health outcomes [1,2,3], which some have attributed to the stability and wealth accumulation afforded by homeownership [4,5]. However, evidence related to how these protective effects on health may vary based on the contexts in which homeownership occurs is limited. There is strong evidence that historical and contemporary racial and ethnic inequities across the United States (U.S.) contribute to differentials in opportunities for homeownership, as well as differentials in characteristics of neighborhoods in which homes are purchased [6,7,8]. Together, these differences raise important questions regarding the extent to which health benefits associated with homeownership may vary across racial and ethnic groups [9,10]. Furthermore, inequities in homeownership opportunities have worsened with the economic recession and the mortgage crisis beginning in 2008, which had particularly adverse effects in the Detroit Metropolitan Area [4]. Specifically, low-income households, which are disproportionately non-Hispanic Black (NHB) and Hispanic residents, have experienced economic strain, reduced home values, and in some cases, foreclosure [11].

In this paper, we examine whether there are variations in health protective effects of homeownership in the DMA, which offers a compelling context for this study for reasons discussed in greater detail below. We further hypothesize that although homeownership may have generally positive effects on health, it may not be equally beneficial for all, especially in neighborhoods with high concentrations of low valued houses. Furthermore, due to the historical and political contexts related to housing that unfolded in the U.S., we hypothesize that neighborhoods with a higher percentage of NHB residents will tend to have lower housing values, and that in neighborhoods with those characteristics, homeownership may be less health protective.

### 1.1. Background and Literature Review

#### Housing and Health

The relationship between housing and health has been examined at both neighborhood and individual levels, with housing location, conditions, and affordability linked to health. Health effects of the geographic location of the home, including for example, variations in the physical (e.g., access to healthy foods, parks, traffic, safety, blight), social (e.g., race-based residential segregation, crime, social capital) and economic (e.g., employment opportunities, investment in educational systems) environments have been documented [12,13,14,15,16]. Other studies have considered the effects of neighborhood conditions on residential stability, measured by homeownership, occupancy status and/or period of tenancy [14,15]. Associations between physical housing conditions and health, include for example, effects of overcrowding, dampness, mold, toxics, and energy efficiency on asthma [17,18], heart disease [19,20], and mortality [21,22]. Housing affordability have been linked to instability, foreclosures and evictions, with consequences for the mental and physical health of those affected [4,23,24,25,26,27]. Furthermore, housing is considered to provide access to political, social, economic and cultural opportunities, all of which are important determinants of health [28].

The preponderance of findings from this body of research conclude that access to affordable, stable and adequate housing is an important determinant of health. However, there has been relatively little consideration of how these patterns may vary across historical, political, economic or geographic contexts. In particular, we focus on historical and political contexts within the U.S., which have led to variations in opportunities for homeownership by race. Our interest is in examining the implications of these racial differences in homeownership for health. 

Narratives in the U.S. link owning a home with having a successful life, often associated with greater security, stability, opportunity and the accumulation of wealth [2,6,28]. Homeownership, home equity and credit can provide opportunities and financial resources for home owners to get ahead [2,29]. In addition, homeownership and the residential stability it provides, have been linked to positive childhood development including stronger vocabulary skills and greater educational attainment [3]. Indirectly, place-based research has considered homeownership and its effects on health at the neighborhood level including the connection between homeownership, neighborhood socioeconomic status and health [30,31], and the increased likelihood for homeowners to invest in the social and physical characteristics of their neighborhoods with positive effects on resources like schools, security and social cohesion [2,16].

Most of the evidence described above has been developed by studying differences in housing tenure (home ownership vs. renters): Comparisons considering health, including mortality rates and health service usage, generally find more favorable outcomes for homeowners [5,32]. These tenure differences have informed the creation of programs like the 2000 Fannie Mae initiative “American Dream Commitment”, which invested trillions of dollars into home financing for affordable housing and opportunities for home purchases by low-income populations [6]. By 2002, approximately 68% of households in the U.S. owned their homes compared to 64% in 1994, and homes purchased by low-income families, NHBs, Hispanics and other labeled racial or ethnic groups (e.g., American Indian Asian, Native Hawaiian), increased faster than other groups [6,33]. Through increased homeownership among low-income households, social, health and economic factors were expected to improve [6,34].

For generations prior to this program, the U.S. government implemented a variety of policy initiatives and market interventions that directly and indirectly expanded the opportunity of homeownership [6,35]. Perhaps most notably, the Federal Housing Administration (FHA) of 1934 was created with the intention of making homeownership more affordable and accessible to middle and low-income households by guaranteeing payment in the case of a default [6,35]. Through the establishment of Fannie Mae, Freddie Mac and other government programs, federal interventions in the housing policy and real estate led to mortgages and land contracts that would expand homeownership across different classes [6]. However, the reality of this expansion was not equally accessible to everyone, as the FHA explicitly used factors like race as a criterion in determining mortgage eligibility [35]. As a result, racial inequities in regards to mortgages and loans emerged [6]. This practice, combined with the practice of redlining, which denied or limited mortgage availability based on the racial or ethnic composition of the neighborhood, contributed to declines in home values in urban communities and restricted housing choice for NHB residents [36].

To address these challenges, the Fair Housing Act of 1968 was implemented to protect residents from discrimination based on race, color, national origin, religion, sex, disability and the presence of children, when renting, buying or seeking financing for housing [37,38]. However, despite its implementation, racial and socioeconomic patterns in access to housing persist [32,37]. There is substantial evidence that both housing policies and real estate markets have had profound impacts on residential patterns over the past century [7,36,39]. Furthermore, the legacy of redlining continues through illegal practices in the housing market including predatory lending and real estate steering [37]. As a result, although homeownership has increased among previously excluded groups, patterns of racial and ethnic segregation have not change substantially [11,40,41]. 

The adverse effects of these policies and practices disproportionately affect NHB, Hispanic and other labeled racial or ethnic groups, and contribute to continued inequities in access to employment and educational opportunities [7,42]. Although race- and class- based residential segregation have been firmly linked to inequities in social and physical environmental conditions that underlie persistent inequities in health outcomes [30,31,43], very few studies consider race- and class-based health inequities within the homeowner population. Consistent with other determinants of health, which are often studied by comparing the presence or absence of the determinant (e.g., employed versus unemployed, high school diploma versus no high school diploma), there continues to be limited research on the potential race, ethnic and class-based inequities that may exist among and between homeowners. This leads to questions regarding variations in the experience of homeownership itself, which could challenge the concept that owning a home is good for your health.

Understanding potential differences in the experience and health implications of homeownership requires examination of policy, community and household level factors as they operate independently and collectively to affect health. In 2014, Novoa and colleagues presented a framework discussing the effects of housing systems, defined by housing policies and the real estate market, as drivers of housing affordability and conditions [44]. They postulate that individual well-being is determined by a combination of neighborhood social and physical factors, as well as dimensions of inequality such as race, age, gender and social class. Although the Novoa and colleagues’ framework does not specifically encompass homeownership, we find it a useful framework for understanding factors that may shape the supply and demand of affordable housing, ultimately influencing patterns of homeownership and variations in the characteristics of the neighborhoods in which homes are purchased. This shift to understanding the upstream determinants of patterns of homeownership is reflected in a shift from the traditional approach of understanding how ‘where you live matters’ to understanding ‘why you live where you live’. Such an approach also allows for the exploration of residential patterns that persist in many U.S. cities like Detroit, Michigan, brought about by historical and political contexts that have framed both housing policies and the real estate market.

Framing housing explicitly as a public health issue, in the context of a health equity framework, enables examination of the role of housing policies and the real estate market on health inequities. This then offers opportunities to identify interventions to address inequities in access to adequate and affordable housing, and ultimately to reduce inequities in population health. 

### 1.2. Conceptual Model of Homeownership, Health and Health Inequities

**Figure 1:**
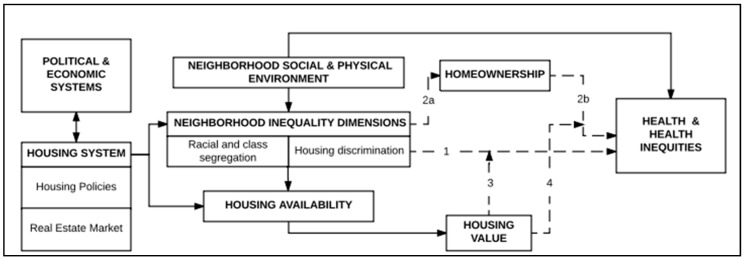
Conceptual model exploring the systemic effects on the relation between homeownership, health and health inequities. The dotted arrows and corresponding numbers represent the pathways examined in the study. Adapted from Novoa et al. 2014 [44]; Saegert et al. 2011 [45].

The conceptual model guiding our research was developed grounded in the literature above and existing frameworks [44,45]. As illustrated in Figure 1, this frameworks links homeownership and health inequities to inequalities in underlying political, economic and housing systems.

The dotted arrows represent pathways examined in this study. While effects of political and economic systems (e.g., democratic decision making, market-based economies) on these pathways are included in the model as factors shaping the housing system, in-depth examination of these systems is beyond the scope of this paper (but see Madden & Marcuse 2016 [28]). Rather, our most immediate focus is on the housing system, conceptualized as consisting of housing policies and the real estate market, as it shapes and influences neighborhood inequality dimensions, including for example, racial- and class-based segregation and housing discrimination. These inequalities across neighborhoods, in turn, also affect housing availability as shown in Figure 1. Housing availability and characteristics of the neighborhood physical and social environments in which the housing is located jointly influence housing value. In the conceptual model, housing value reflects both collective effects at the neighborhood level (e.g., neighborhood social status, and investment) and individual level (e.g., housing wealth and affordability) and housing conditions (e.g., the cheaper the house the more potential need for repairs), not pictured here. Although our focus is at the neighborhood level, it is important to note that variations across neighborhoods in housing condition, affordability and physical and social neighborhood conditions are theorized to disproportionately adversely affect labeled racial, ethnic, and lower socioeconomic groups. These dimensions of inequality ultimately translate into the unequal social patterning of health outcomes (see Novoa and colleagues [44]).

While not depicted in this model, context also plays a role in influencing the different pathways and outcomes of our model. With respect to the relationship between homeownership and health, context can include dimensions such as historical events, political climate, economic situations of a city and geographic location [44]. This relation also plays a role in the selection of indicators used to study inequities in homeownership and health. For example, in Spain, studies have focused on social class as the inequity dimension shaped by the economic and political context that has influenced associations between homeownership and health [46]. The historical and political contexts of the DMA, described below, are relevant to this study of race-based inequities shaping the homeownership and health relationship [11]. 

#### Detroit Metropolitan Area Past & Present

The DMA is comprised of Oakland, Macomb and Wayne (where Detroit city is located) counties. In 2010, the population of the DMA was approximately 3.8 million with 18% located in Detroit city [47]. In the early 1900s, the DMA ranked among the nation’s largest metropolises with industrial jobs recruiting residents from the South, Canada and Mexico [11,40]. Numerous factors have contributed to the creation and persistence of racial residential segregation in the DMA, including the consequences of the FHA redlining practices throughout the city [11,31,40]. During the mid-20th century, suburbanization, deindustrialization, and federal transportation policies led many white residents, businesses and economic investments to leave the city for its surrounding suburbs [11,31,40]. In combination, the relocation of employment opportunities and the continuation of exclusionary housing practices exacerbated residential segregation and increased unemployment among NHB residents [11,31,48].

The DMA continues to reflect these historical processes with differences in socio-demographic characteristics among its residents, including race. In Oakland and Macomb Counties, there are approximately 76.5% and 82.9% Non-Hispanic Whites (NHWs), respectively, while in Wayne County 54.8% are NHW with 85% of the NHB population located in Detroit city [49].

The massive disinvestment in Detroit city is also evident in the housing stock including both the lack of investment in affordable housing and current housing conditions. Most houses in Detroit (92.1%) were built prior to 1980 [49,50]. In 2015, the American Housing Survey, a representative longitudinal housing unit survey, reported that in Detroit and the adjacent cities of Warren and Dearborn, 32.3% of households surveyed had an external building problem (e.g., broken windows, roof damage, boarded units), 8.8% had mice, and approximately 13.7% reported being uncomfortably cold during the winter [50].

## 2. Methods

Following the conceptual model presented above, we assess the following specific hypotheses:

- Is neighborhood percent NHB associated with homeownership (pathway 2a)?
- Is percent NHB associated with health outcomes (pathway 1)?
- Are associations between NHB and health outcomes mediated by homeownership (pathway 2a + 2b)?
- Are associations between racial composition and health outcomes modified by neighborhood housing value (pathway 3)?
- Are associations between homeownership and health outcomes modified by neighborhood housing value (pathway 4)?

### 2.1. Data and Measures

The goal of this paper was to assess ecological associations between neighborhood level characteristics. We used data on racial composition, homeownership, housing value and two health outcomes, disability and all-cause mortality, from the DMA for this analysis. Neighborhood level data on racial composition, homeownership and housing value, along with our control variables of neighborhood mean age, education and household income, came from the 2009–2013 (5-year estimate) American Community Survey (ACS), a publicly available yearly survey representative of the population conducted by the U.S. Census Bureau [49]. Mortality data (2009–2013) came from the Michigan Department of Community Health [51]. Data were aggregated to the census tract (CT) level [51] as a proxy for neighborhood to capture the variability across geographic areas within the DMA. This level was selected as the finest spatial scale at which all data used in this analysis were available, and to maximize comparisons with previous research [12,31]. Across the three counties there were a total of 1166 CTs: 15 CTs were eliminated from the final count of 1151 due to insufficient population or data availability.

*Dependent variables* included disability and all-cause mortality as health indicators, at the neighborhood (CT) level. *Disability* was measured using the percentage of individuals aged 18–64 described as having one or more of the following difficulties: hearing, vision, cognitive, ambulatory, self-care, and independent living [52]. *All-cause mortality* was measured as the percent of the total population that died due to any cause per year, averaged for the five-year period, 2009–2013 [51].

*Independent variables* included measures of neighborhood racial composition, homeownership and home value. Neighborhood racial composition was operationalized as *neighborhood percent non-Hispanic Black (NHB)*, constructed using the percentage of NHB residents divided by the total number of residents in the CT. The result was a continuous variable of the percent of NHB residents in the neighborhood. For the *homeownership* indicator, the total number of houses occupied by their owners was divided by the total number of houses occupied (owners and renters) in the CT to provide the percentage of homeowners. The *housing value* indicator used the ACS housing value question, which asked homeowners to estimate the value of their home (house and lot, mobile home and lot or condominium unit) [53]. Although not a perfect measure as it excludes housing values of rented properties, this data provides an approximation that has been widely used to reflect neighborhood quality, wealth and housing affordability to develop housing programs in the area [53]. Our measure of *housing value* used the ACS values to calculate the percent of homes valued at $50,000 or more, constructed by dividing the number of owner-occupied housing units in the CT valued at or above $50,000 by the total number of housing units in the census tract. The cut-off value used in these analyses is slightly higher than the $43,600 median house value in Detroit [54] and lower than the $134,000 Michigan state median house value [55]. The result was also a continuous variable reflecting the percent of homes valued at $50,000 or more in the CT.

Control variables included age, percentage of population in neighborhood aged 18 to 64, education, percentage of population in neighborhood with a high school diploma or more, and median household income. Each of these variables was constructed at the CT level.

### 2.2. Data Analysis

For the total sample and subsamples representing each county, we described and compared the neighborhood (CT) mean and standard deviation for all variables included in the study. Multiple regression models were used to test our hypotheses. To test our first hypothesis, we regressed neighborhood percent homeownership on neighborhood percent NHB (pathway 2a). To assess our second hypothesis (pathway 1), we regressed disability and all-cause mortality outcomes on neighborhood percent NHB, controlling for age and education (models 1a and 1b). We then added homeownership (models 2a and 2b) and using the Sobel test, we completed a mediation analysis to examine the effects of homeownership on associations between NHB and health outcomes (pathway 2a, 2b). We also assessed sensitivity of these models with inclusion of neighborhood income. Next, we added interaction terms (1) between housing value and racial composition (pathway 3) to assess the extent to which housing value modified associations between NHB and health (models 3a and 3b) and (2) between housing value and homeownership (pathway 4) to assess whether housing value modified the association between homeownership and health outcomes (models 4a and 4b). All statistical analyses were completed using SAS 9.1 (SAS Institute Inc., Cary, NC, USA, 2002–2003). Results are described below.

## 3. Results

In Table 1, we present the descriptive statistics for the full sample (1151 CTs) and each county, including the 5-year mean and standard deviation at the neighborhood level for the DMA between 2009 and 2013. 

The mean percent of residents aged 18–64 for census tracts within the DMA was 63%. On average, approximately 86% of residents at the CT level completed high school. The average neighborhood percent NHB was 31%, ranging from 10% in Macomb, 14% in Oakland and 48% in Wayne County. The mean percent of homeowners at the CT level was 68%, while neighborhoods in the study area had a mean of 76% of homes valued at $50,000 or more. For health outcomes, the average percentage of individuals aged 18–64 with a disability at the CT level for the full sample was 14%, while the all-cause mortality average was 951.44 (per 100,000).

Percent NHB was inversely correlated with the percent of housing valued above $50,000 (Pearson X^2^ = 262.02, *p* < 0.001) median household incomes (Pearson X^2^ = 282.13, *p* < 0.001), and neighborhood homeownership (Pearson X^2^ = 108.52, *p* < 0.001). Neighborhood percent housing valued greater than $50,000 was positively correlated with median household incomes (Pearson X^2^ = 220.66, *p* < 0.001) (results not shown).

Results from tests of our first hypothesis, that neighborhood percent NHB was associated with homeownership, found a significant inverse association, after accounting for neighborhood mean age and education (B = −0.22, *p* < 0.001). These associations were reduced (B = −0.12, *p* < 0.001) but remained significant after controlling for neighborhood median income (results not shown).

Results from tests of our second hypothesis (Table 2) show significant associations between neighborhood percent NHB and disability (Model 1a, B = 0.097, *p* < 0.001) and all-cause mortality (Model 1b, B = 2.748, *p* < 0.001). Models incorporating median household income as a control variable indicated that associations between percent NHB and disability (B = 0.073, *p* < 0.001) and all-cause mortality (B = 0.75, *p* = 0.016) were reduced but remained statistically significant (results not shown).

Results from tests of our third hypothesis indicate the homeownership was inversely associated with disability (Model 2a, B = −0.023, *p* < 0.05) and all-cause mortality (Model 2b, B = −3.783, *p* < 0.001). In these models, associations between percent NHB decreased but remained significant for both disability (B = 0.094, *p* < 0.001) and mortality (B = 1.854, *p* < 0.001). Due to multicollinearity, we do not report results for models incorporating mean neighborhood income. Results from Sobel tests to assess the hypothesis that homeownership mediates associations between percent NHB and health outcomes indicate that the association between percent NHB and mortality was partially mediated by homeownership (z = 2.86; *p* < 0.05), but not for disability (z = 0.42; *p* > 0.1) (results not shown).

Results from tests of our fourth hypothesis, that associations between neighborhood percent NHB and health outcomes are modified by neighborhood housing values are shown in Table 3, models 3a and 3b. We found no support for the hypothesis that neighborhood housing value modified associations between neighborhood percent NHB and disability (B = −0.000, *p* > 0.1) or all-cause mortality (B = −0.011, *p* > 0.1). Results from tests of our fourth hypothesis (Table 3, Models 4a and 4b) suggest that neighborhood housing value significantly modified associations between homeownership and both disability (B = −0.001, *p* < 0.001) and all-cause mortality (B = −0.075, *p* < 0.001).

**Figure 2:**
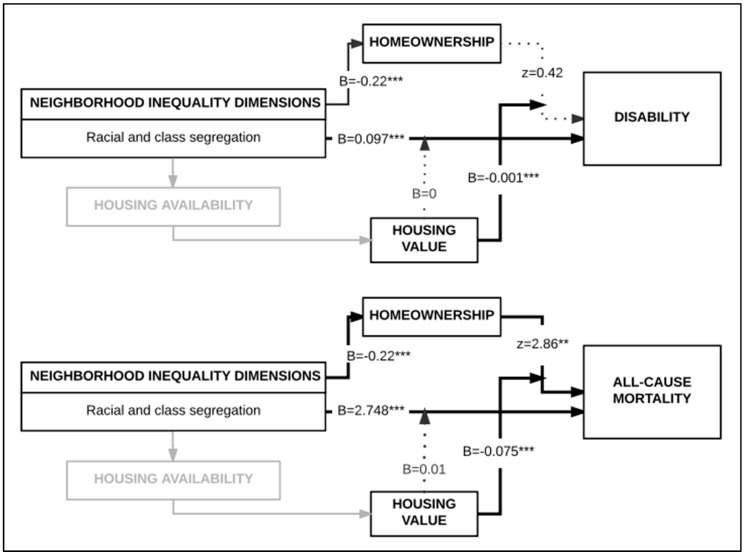
Pathway results examining the relation between neighborhood racial composition, homeownership and housing value for each of the two health outcomes, disability and all-cause mortality. Only tested pathways are illustrated in black. B values are coefficient estimates; z values are Sobel test results; ** *p* < 0.05; *** *p* < 0.001.

Based on these results, we revisited our conceptual model. The revised model is shown in Figure 2 for each health outcome. *Housing availability* was kept in the model due to its conceptual importance but because it was not directly tested in these models, appears in light grey.

## 4. Discussion

There were four major findings from our tests of pathways linking racial and socioeconomic inequities, home ownership, and health. First, neighborhood percentage NHB was significantly and inversely correlated with neighborhood homeownership. Second, neighborhood percentage NHB was inversely associated with both disability and all-cause mortality, an association that remained significant after accounting for neighborhood household income. Associations between percentage NHB and health outcomes were partially mediated by homeownership for mortality but not for disability. Third, we found no evidence that neighborhood housing values modified associations between neighborhood percent NHB and either of the health outcomes included in these pathways. Finally, our findings suggest that neighborhood housing values significantly modify associations between home ownership and health outcomes, with homeownership in neighborhoods with higher house values more strongly associated with reduced disability and all-cause mortality. We discuss these findings and their implications in greater detail below.

*Is neighborhood racial composition associated with homeownership rates?* We found significant bivariate correlations between neighborhood percent NHB and homeownership, and these associations remained significant after accounting for neighborhood mean age, mean education and median household income. Neighborhood percentage NHB was also significantly and inversely correlated with home values and household income. It is likely that in highly segregated areas such as the Detroit Metropolitan Area, these correlations reflect a combination of discriminatory housing policies, and discriminatory employment policies (contributing to lower incomes) [11], ultimately leading to fewer homeownership opportunities within predominantly NHB neighborhoods.

*Are associations between neighborhood racial composition and health outcomes mediated by homeownership?* Our finding of a significant association between neighborhood percent NHB and health outcomes is consistent with results from numerous studies that have shown neighborhood health inequities based on the racial and ethnicity composition of neighborhoods [9,56,57,58]. Given the history of racial and ethnic discrimination in housing markets in Detroit [11], including racial differences in opportunities for homeownership [11,41] and house values [48,59], we specifically examined the extent to which this association was mediated by homeownership, or modified by neighborhood housing values. Our findings suggest that homeownership was protective against both disability and all-cause mortality, and significantly mediated associations between neighborhood percent NHB and all-cause mortality, but not disability. Associations between percentage NHB and mortality were partially explained by variations in homeownership across neighborhoods. Additional research examining the causal pathways linking neighborhood percent of NHB with reduced homeownership, and ultimately increased mortality, will be critical to intervening in this process to reduce health inequities. Associations between racial composition and all-cause mortality were significantly mediated by neighborhood level homeownership. Associations between racial composition and disability were not significantly mediated by homeownership. Further exploration of pathways linking these variables is needed.

*Are associations between neighborhood racial composition and health outcomes contingent upon neighborhood home values?* We did not find support for the hypothesis that associations between neighborhood percent NHB and either disability or all-cause mortality were modified by neighborhood housing values. In other words, regardless of housing value, the neighborhood percent NHB was positively associated with disability and all-cause mortality. Future studies with access to individual-level data could provide further exploration on the relationship between race, socioeconomic status and health.

*Are associations between home ownership and health outcomes contingent upon neighborhood house values?* Our findings indicated housing value did moderate this relationship, meaning that homeownership in neighborhoods with a greater proportion of housing valued at $50,000 or more were more strongly protective of health than in neighborhoods with a smaller proportion of homes valued at $50,000 or more. This effect was evident both for disability and for all-cause mortality. Studies have used similar measures of housing value that encompass respondents’ input on neighborhood conditions and recent sales of adjacent homes [53], and have linked low housing values to greater vacancy and blight [60,61], reduced safety and security [62], and decreased investment in neighborhood resources like education and employment opportunities [63], all of which have been linked to negative health outcomes [16,45,64]. In the DMA, respondent self-reports of their home values during the post-recession period would also reflect poor economic conditions across the region, and especially in Detroit. The DMA had one of the highest subprime market penetration rates in the country, and in 2005, 68% of all mortgages in the city of Detroit were subprime, compared to 24% nationwide [65]. By 2015, more than half of foreclosed homes were blighted, needed demolition, or had been foreclosed for nonpayment of taxes [65]. Victims of subprime lending and homeowners in neighborhoods hardest hit by the effects of the foreclosure crisis would not only experience poorer health due to deteriorating neighborhood conditions, but also the effects of *negative equity*, where the market value of the property falls below the outstanding mortgage balance, resulting in no path to debt reconciliation [66,67]. By eliminating racial composition from the analysis, we observed greater variation in percentages of homeownership and housing values across neighborhoods, allowing for a closer examination of the moderating effects of housing value on the relation between homeownership and health.

### 4.1. Limitations

The research presented here is an initial step towards better understanding inequities in homeownership and their relationship to health outcomes. However, there are some limitations in data availability and analysis. Tests of pathways in the models presented in this paper focus on neighborhood level (census tract) associations. Our findings suggest that residents of neighborhoods with higher proportions of NHB residents have lower home values and lower rates of homeownership and thus derive fewer health benefits than residents of neighborhoods with lower proportion of NHB residents. These models do not allow analyses of individual-level dimensions. Thus, we are not able to examine, for example, whether benefits derived by NHB homeowners in high income neighborhoods are greater than those derived by NHB homeowners in lower income neighborhoods, or whether NHB and NHW homeowners in higher income neighborhoods derive similar benefits. Future studies should involve tests of these associations using multilevel analyses that account for individual level characteristics. There may be additional confounders that were not accounted for in this analysis (e.g., pre-existing disease, behavioral risk factors), resulting in issues of residual confounding and potential bias [68]. Future analyses that account for additional individual level or community level factors potentially associated with the dependent variables will be important to further assess the associations reported in this paper. Furthermore, the cross-sectional nature of the ACS data used in these analyses does not allow for past comparisons of health conditions to determine any changes in outcomes. Future studies with access to longitudinal data can consider trends in health inequities over a longer time period to also capture the effects of political and economic events like the economic crisis and foreclosures on health outcomes. Tests of mediation and modification would be similarly strengthened using longitudinal data. Results of our test for mediation should be interpreted with caution due to the cross-sectional nature of the data [69]. Analyses of cross-sectional mediation pathways can suggest possible causal mechanisms, particularly when the interpretation of cross-sectional measures is informative about the temporal process. It is plausible that results from our test for mediation using cross-sectional data provides biased estimates [70]. The finding of a significant mediation effect using cross-sectional data in this instance suggests that future analyses using longitudinal data to explore this mediation may be fruitful.

In addition, due to cost limitations associated with accessing real estate data, our housing value indicator was limited to responses from home owners [53] as provided by the ACS. Together with the disability variable, another self-reported indicator, potential same source bias could arise with respondents who live in neighborhoods with lower valued homes, reporting more disabilities, or those in higher house valued neighborhoods reporting better health. Nonetheless, the indicator still provides some input on neighborhood conditions and mimics the housing value assessment process done by property assessors to estimate values based on similar properties in the neighborhood, minus the depreciation of the house and land value [71]. Future studies could address such issues by incorporating housing value data for all households at the CT level, if available, or using longitudinal studies that can control for health outcomes at baseline.

### 4.2. Contributions and Next Steps

Despite these limitations, this study provides a conceptual model and examines empirical evidence that connects homeownership to health outcomes. We provide evidence that homeownership is protective of health, but that opportunities for these health protective effects are not equitably distributed within residentially segregated areas such as the DMA. Specifically, findings from text of our first and second hypotheses suggest that in neighborhoods with higher proportions of NHB residents, there are fewer opportunities for residents to experience protective effects of homeownership. Furthermore, our finding that protective effects of homeownership are contingent upon median home values, and that these differ based on racial composition of the neighborhood, suggest that homeownership may be less protective of health in neighborhoods where home values are lower, and these are disproportionately neighborhoods with higher percentages of NHB residents. We have begun to disentangle race and socioeconomic status in these models, but future efforts are needed to understand more clearly the association between race, socioeconomic status and health outcomes and the pathways through which these health effects occur. Furthermore, these findings reflect the historical and political contexts that have shaped this post-industrial city and thus may differ in other metropolitan areas.

Our findings contribute to efforts to conceptualize housing explicitly as a public health issue in the context of a health equity framework. By illustrating significant correlations between housing variables and health outcomes, and testing potential mediating as well as modifying pathways, this study lays a foundation for future analyses that further test these associations. Developing a solid evidence base linking housing access to health outcomes provides critical information with which to inform policy change to address the contributions of housing to health inequities. Future studies are needed to continue building evidence in this area and go beyond to raise questions on what types of housing conditions (e.g., affordability, physical and social to name a few) and under what contextual circumstance does homeownership influence health. In the case of the DMA, historical and political events have shaped neighborhood racial compositions, and affected housing values, resulting in homeownership health inequities. Nonetheless, the effects of these events persist, and with every new wave of economic adversity, declining housing values threaten to perpetuate these inequities [4]. Therefore, in order to promote equity in homeownership and health, policies and interventions should implement strategies to recover and preserve housing values in neighborhoods most vulnerable to economic decline [4,46,64].

## 5. Conclusions

This paper contributes to the evidence needed to develop scientifically informed intervention and policy recommendations that translate this research into actions to reduce health inequities. In this study, we have presented a conceptual model and provided empirical evidence to test whether homeownership is always protective of health outcomes. Although our findings supported our hypothesis demonstrating health inequities in homeownership based on neighborhood racial composition and housing values, more evidence is needed. Future studies should consider other inequity dimensions including class, gender, age, and ethnicity, while also considering the historical and political factors that have shaped the housing system. Such an approach provides a valuable departure from traditional studies that have largely examined the impact on health status for renters compared to homeowners. It also provides the evidence needed to inform and implement policies and interventions aimed at reducing homeownership health inequities.

## Figures and Tables

**Table 1:** Sample mean and standard deviation (SD) for all study variables in the DMA at the neighborhood level, 2009–2013.

	Total Sample Mean (SD) (N = 1151 CT)	Oakland Mean (SD) (N = 337 CT)	Macomb Mean (SD) (N = 212 CT)	Wayne Mean (SD) (N = 602 CT)
Demographics				
% 18–64 years	62.86% (5.57)	63.41% (5.76)	62.86% (4.01)	62.55% (5.90)
% high school diploma	86.27% (10.22)	92.53% (6.17)	87.81% (5.22)	82.22% (11.39)
% Non-Hispanic Black (NHB)	30.94% (37.03)	14.21% (22.31)	10.15% (11.95)	47.63% (41.32)
Median household income (in thousands)	$52,000	$72,000	$53,000	$42,000
Housing Characteristics				
% homeowners	67.71% (21.51)	72.80% (22.09)	75.43% (16.72)	62.14% (21.15)
% housing value at or above $50,000	76.25% (23.58)	89.85% (14.12)	83.99% (16.80)	65.94% (24.94)
Health Outcomes				
Disability (% aged 18–64 with one or more disability)	13.48% (7.71)	8.92% (5.22)	11.99% (5.25)	16.56% (8.17)
All-Cause Mortality (per in 100,000 population)	951.44 (369.99)	770.41 (298.51)	1004.02 (333.91)	1034.26 (383.10)

**Table 2:** Neighborhood level disability and all-cause mortality regressed on percent NHB (Models 1a and 1b), and homeownership (Models 2a and 2b).

	Disability (% Aged 18–64 with One or More Disability)				All-Cause Mortality (per in 100,000 Population)			
	Model 1a		Model 2a		Model 1b		Model 2b	
	Estimate	Std. Err.	Estimate	Std. Err.	Estimate	Std. Err.	Estimate	Std. Err.
Percent NHB	0.097 ***	0.005			2.748 ***	0.391		
Percent NHB			0.094 ***	0.005			1.854 ***	0.421
Homeownership			−0.023 **	0.009			−3.783 ***	0.777

** *p* < 0.05; *** *p* < 0.001; All models were controlled for neighborhood mean age and mean; Std. Err., Standard Error education.

**Table 3:** Modification of the association between percent NHB and health outcomes (Models 3a and 3b) and homeownership and health outcomes (Models 4a and 4b) by neighborhood housing value.

	Disability (% Aged 18–64 with One or More Disability)				All-Cause Mortality (per in 100,000 Population)			
	Model 3a		Model 4a		Model 3b		Model 4b	
	Estimate	Std. Err.	Estimate	Std. Err.	Estimate	Std. Err.	Estimate	Std. Err.
Percent NHB	0.062 ***	0.014			3.489 ***	1.039		
Housing Value	−0.079 ***	0.014			0.461	1.021		
Percent NHB * housing value	0.000	0.000			−0.011	0.015		
Homeownership			0.013	0.028			2.465	1.910
Housing Value			−0.091 ***	0.021			1.670	1.448
Homeownership * housing value			−0.001 **	0.000			−0.075 ***	0.022

* *p* < 0.1, ** *p* < 0.05; *** *p* < 0.001; All models were controlled for age and education; Std. Err., Standard Error.
